# Supplementary material for: Establishment of an antimetabolite-based transformation system for the wood-decaying basidiomycete Phanerochaete chrysosporium
Source: Appl Environ Microbiol. 2025 Sep 5;91(10):e01160-25. doi: 10.1128/aem.01160-25 (PMC12542683; doi:10.1128/aem.01160-25)

Supplemental material

of

Establishment of an antimetabolite-based transformation system for the wood-decaying basidiomycete *Phanerochaete chrysosporium*

Kazuma Masumoto[1#], Petra Banko[2#], Ayane Yamamoto[2], Kyoko Miwa[1, 2], Chiaki Hori[1, 2]*

[1]Course in Molecular Biology, Division of Biosphere Science, Graduate School of Environmental Science, Hokkaido University, Sapporo, Japan

[2]Environmental Molecular Biology, Section of Environmental Biology, Faculty of Environmental Earth Science, Hokkaido University, Sapporo, Japan

# These authors contributed equally.

*Address correspondence to Chiaki Hori, chori@ees.hokudai.ac.jp

Table S1. Inhibitory effect of PT concentration on protoplast recovery of the wild-type *P. chrysosporium*. Approximately 2 × 10^6 protoplasts were spread on CD agar plates supplemented with 0, 0.1, 0.3, 0.5, and 1.0 µg/mL PT and cultured for 4 days at room temperature. Colony numbers were counted.

**PT conc. (µg/ml)**	0	0.1	0.3	0.5	1.0
**Colony number**	41	10	9	8	4

Table S2. Primers used in this study

**Primers**	**Sequences (5' to 3')**
pPTRII-For	ACTGGCCGTCGTTTTAC
pPTRII-Rev	TTTGTGATGCTCGTCAGGGGGG
ITS1-For	CTTGGTCATTTAGAGGAAGTAAAAGTCGT
ITS4-Rev	TCCTCCGCTTATTGATATGC
eGFPcd_For	GGTTTCCAAGGGCGAAGAGC
eGFPcd_Rev	CGTCCTTGAAAAAGATGGTT
*PchGAPDH*pro(1140)_KpnI_For	GGTACCCATCGGCAGGCCTTGGC
*PchGAPDH*pro(1740)_KpnI_For	GGTACCCGTGTTCCGCGACGAACC
*PchGAPDH*pro_SbfI_Rev	CCTGCAGGGTTCAAGTAGTGTAGGGGTG
*Pchactin1*pro_KpnI_For	GGTACCCCGGTGTCACGCGCAAGG
*Pchactin1*pro_SbfI_Rev	CCTGCAGGTGTAGTGTACTAGTTGGTTGGG
*PchDED1*pro_KpnI_For	GGTACCAGGCATGTCAACCTCGTTGAC
*PchDED1*pro_SbfI_Rev	CCTGCAGGATTAAGGGGAATGAGAAGTGG
*actin1*_atgg_Sbf1_Rev	CCTGCAGGGGCCATTGTAGTGTACTAGTTGGTTG
*actin1*_eie_Sbf_Rev	CCTGCAGGCTGCTTTGACCTGGAAAGCGAAGTCAGCACGCGGTCGTCGC GGTCGGGCTGTGTGGTGTACTGACCGGCATTGTAGTGTACTAGTTGGTTG
AMA1_Rep2_For	CCTCGGGCTTTCCCCGTCAAGCTC
AMA1_UCS_Rev	AAAGCCCGAGGAACGGCGGAATGC

Figure S1. Genomic PCR analysis of PT-resistant colonies of *P. chrysosporium* transformed with GFP expression vectors containing the *GAPDH*1140, *GAPDH*1740, *DED1*, and *actin1* promoters referred to in Figure 2. The presence of the vector was confirmed by amplifying the GFP coding region from the genetic extracts of 16 resistant colonies. The ITS region was amplified and used as a positive control and reference for PCR conditions. (PC: pPTRII positive control, NC: negative control, WT: wild type)

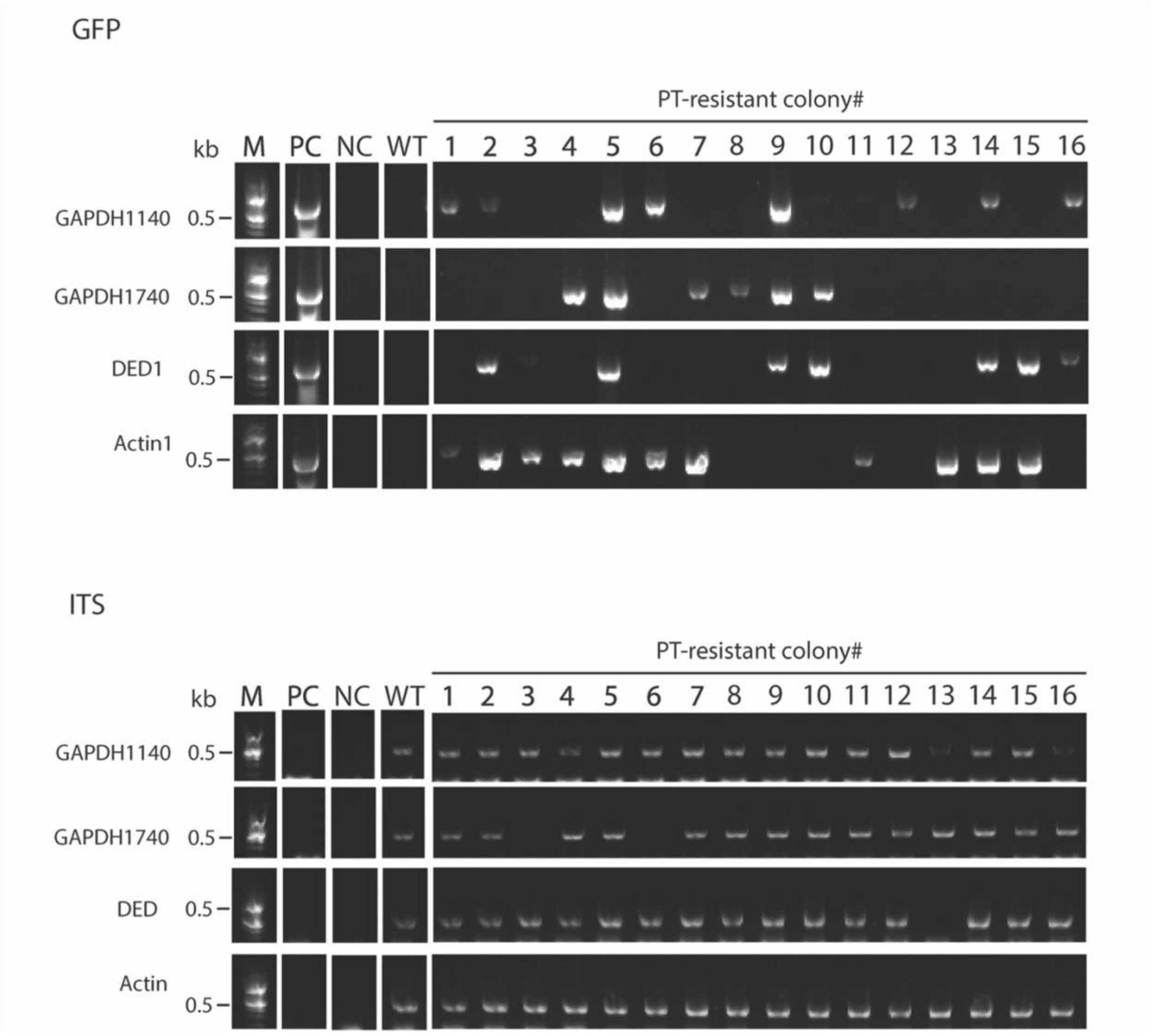

Figure S2. Genomic PCR analysis of PT-resistant *P. chrysosporium* colonies transformed with the respective modified GFP expression vectors referred to in Figure 3. The presence of the vector was confirmed by amplifying the GFP coding region from the genetic extracts of 16 resistant colonies. The ITS region was amplified and used as a positive control and reference for PCR conditions. (PC: pPTRII positive control, NC: negative control, WT: wild type)

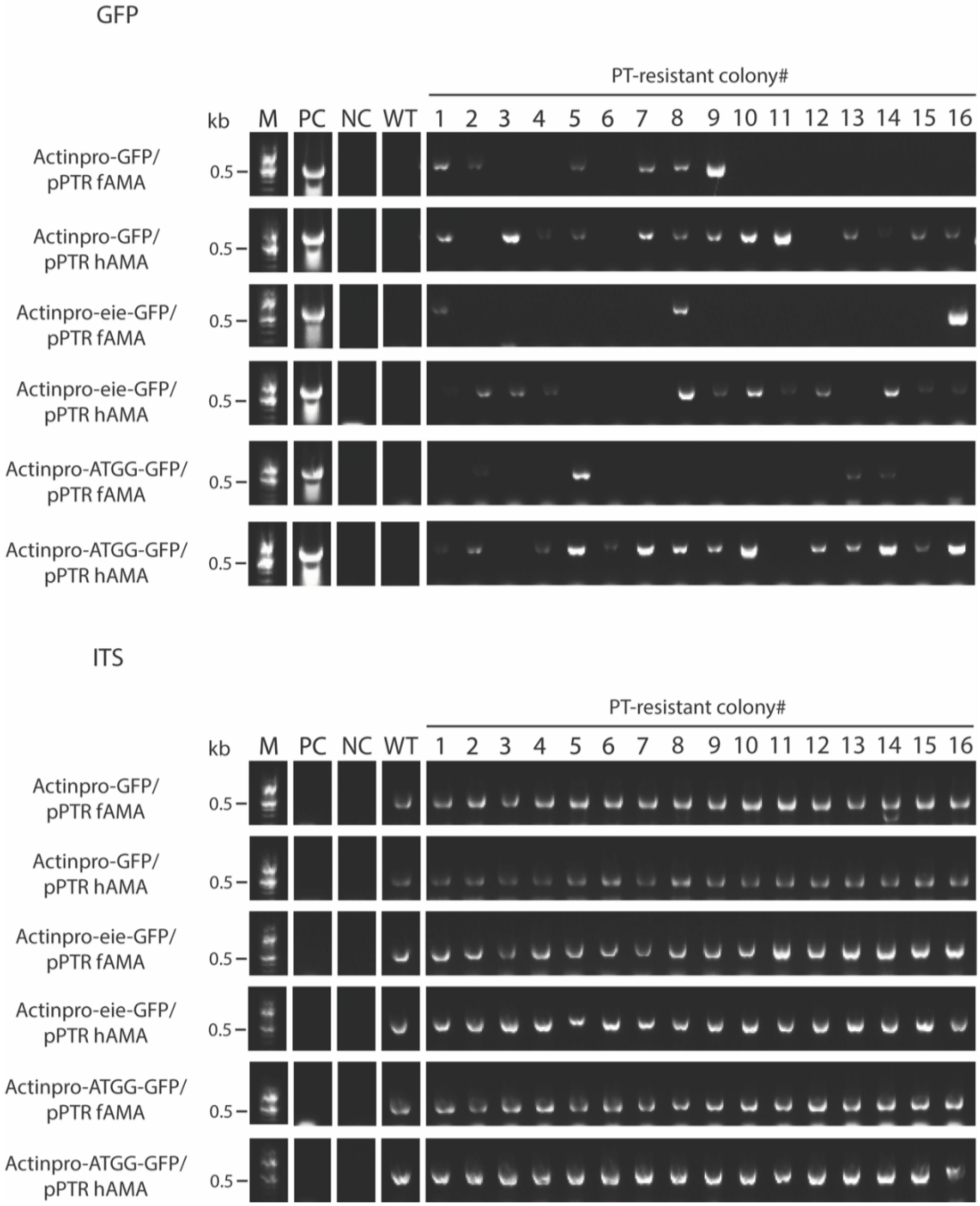

Figure S3. GFP fluorescent signal is distributed throughout the cytoplasm, except for the vacuoles, as demonstrated by enlarged microscopic images of the mycelia transformed with the pPTRII-hAMA1-*actin1*proGFP. The left panel shows the bright field, and the right panel shows the fluorescent micrographs of transformed mycelia. Overlay images reveal GFP fluorescent loss in vacuoles, as indicated by white arrows.

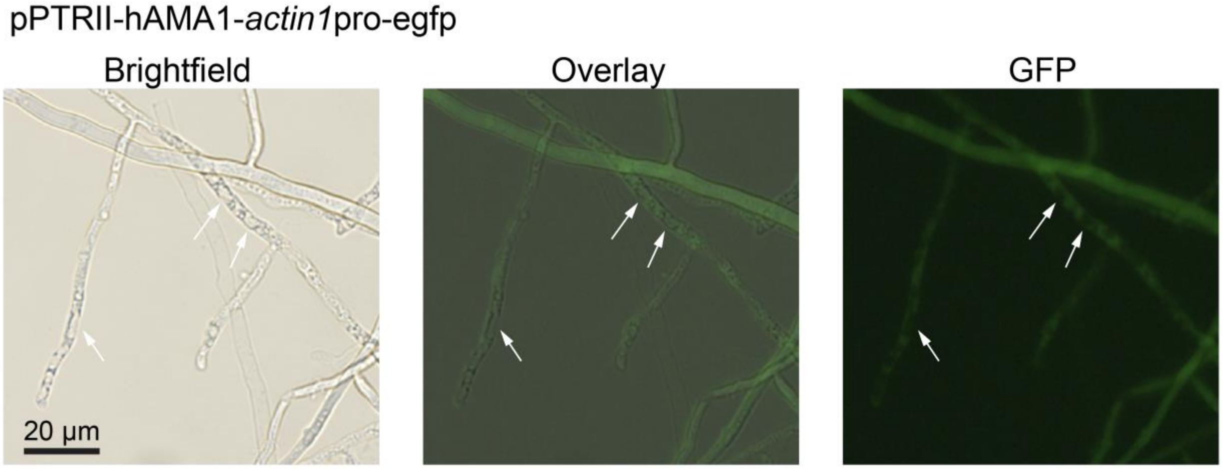

Supplement: Supplemental material — Tables S1 and S2; Fig. S1 to S3. [file aem.01160-25-s0001.pdf]
